# Implementation and Evaluation of the Stroke Triage App Among Paramedics in Prehospital Emergency Care: Sequential Mixed Methods Study

**DOI:** 10.2196/105987

**Published:** 2026-11-03

**Authors:** Femke Kremers, Ellen Hu, Milou Silkens, Naomi de Ruijter, Ruben M. van de Wijdeven, Esmee Venema, Henk Kerkhoff, Sandra Sülz, Bob Roozenbeek, On behalf of the PRESTO-2 Investigators, Jasper van Duijvenbooden

**Affiliations:** 1 Department of Neurology, Erasmus MC University Medical Center, Rotterdam, Zuid-Holland, The Netherlands; 2 Department of Health Services Management & Organization, Erasmus School of Health Policy & Management, Erasmus University Rotterdam, Burgemeester Oudlaan 50, Rotterdam, Zuid-Holland, 3062 PA, The Netherlands, 31 0619673391; 3 Department of Emergency Medicine, Erasmus MC University Medical Center, Rotterdam, Zuid-Holland, The Netherlands; 4 Department of Neurology, Albert Schweitzer Hospital, Dordrecht, Zuid-Holland, The Netherlands; 5 See Acknowledgments

**Keywords:** telehealth usability, stroke, prehospital triage, mixed methods, impact, implementation, telehealth tools, digital health technology

## Abstract

**Background:**

Digital decision-support tools, such as the Stroke Triage App (STA), may enhance prehospital triage and clinical decision-making for suspected patients with stroke. However, implementation of decision-support tools and their impact on clinical practice remains insufficiently studied.

**Objective:**

This study aimed to investigate paramedics’ experiences with the implementation of the STA in prehospital practice by evaluating the implementation process, the implementation outcomes, and its impact on clinical workflows, while considering contextual factors that may have influenced these dimensions.

**Methods:**

STA implementation was evaluated among paramedics in 2 emergency medical services regions in the Netherlands using a sequential mixed methods approach. Actual usage data were extracted from the STA dashboard. Usability, perceived impact, and contextual factors were explored using an online survey and semistructured interviews.

**Results:**

Monthly triages ranged from 52 to 153 (median 120, IQR 107‐131). Paramedics’ individual STA use ranged from 1 to 47 times (median 7, IQR 3‐13). Out of 246 paramedics with an account, 76 (31%) completed the survey, and 9 (4%) participated in semistructured interviews. The STA was generally well accepted, supporting structured neurological assessment and communication. Swift technical support, frequent promotion, and use of local ambassadors facilitated STA use. Clinical urgency, poor workflow integration, and limited perceived added value hampered STA use. Users recommended improvements in mobile interface, login features, and additional explanation with triage advice.

**Conclusions:**

This study demonstrates that the STA achieved substantial reach and initial adoption within the prehospital setting. However, widespread acceptance did not consistently translate into routine integration in daily practice. Successful implementation depends as much on addressing organizational and human factors as on technical performance.

## Introduction

Digital health technologies are increasingly developed and introduced into health care systems to support clinical decision-making, enhance efficiency, and improve patient outcomes [1]. To date, research has predominantly focused on the technical validation of these tools, often underexploring the broader sociotechnical dimensions that determine implementation success [2]. Consequently, translating technological promises into real-world clinical impact remains challenging, as the complex interplay of organizational, clinical, and human factors often influences success beyond mere technical performance [3].

This challenge is particularly evident in the setting of prehospital emergency care, where paramedics must make time-critical decisions under rapidly changing clinical and logistical circumstances [4]. When introducing digital decision-support tools in this setting, specific barriers often emerge. For instance, the time investment required for data entry and device interaction creates a burden that competes directly with urgent patient care [5]. Furthermore, technical limitations, such as unreliable connectivity or environmental constraints limiting device usability, frequently disrupt established clinical workflows [6]. Beyond these practical hurdles, concerns over professional autonomy often hinder trust [7]. Paramedics may perceive such tools as a threat to their expertise, particularly when recommendations lack transparent reasoning or fail to account for the intuitive judgment required in the field.

Such challenges are especially relevant for acute ischemic stroke, where the time sensitivity of reperfusion therapies underscores the negative impact of any technical or operational delay [4]. Traditionally, stroke care has followed a “drip-and-ship” model, with patients transported to the nearest hospital regardless of stroke subtype [8]. However, as eligibility for endovascular treatment (EVT) continues to expand, the need for rapid triage of patients with a suspected large vessel occlusion (LVO) to EVT-capable hospitals has become increasingly urgent [9]. Since treatment delays are strongly associated with poorer patient outcomes, optimizing prehospital triage has emerged as a key strategy to address the logistical challenges of regional stroke networks, thereby reducing unnecessary transfers and accelerating EVT for eligible patients [10].

Within the Dutch health care network, the proximity of general hospitals and EVT-capable hospitals poses a triaging dilemma. Paramedics must balance the benefit of immediate intravenous thrombolysis (IVT) at a nearby general hospital against the risk of delayed EVT or the need for secondary transfer [11]. This decision is further complicated by the diagnostic uncertainty of prehospital screening tools and unknown hospital workflow times [12]. To optimize prehospital triage, digital decision-support tools such as the Stroke Triage App (STA) have been developed and introduced by a multidisciplinary team of clinical stroke experts, paramedics, and technical developers [11,13]. With the aim to assist paramedics in the complex decision-making process, the app integrates neurological clinical assessment via the Rapid Arterial Occlusion Evaluation (RACE) score (used to estimate the probability of an LVO) with contextual data, including symptom onset time, real-time GPS-based driving times, time-dependent treatment effects, and hospital workflow metrics [11-13]. The app combines these variables to estimate the probability of a good functional outcome at 3 months and recommends the optimal transport destination to the paramedic: the nearest general hospital or direct transfer to a more distant EVT-capable hospital.

Achieving the clinical value of the STA and ultimately improving patient outcomes requires not only technical accuracy but also successful implementation, integration into clinical workflows, and acceptance by paramedics, within the context in which the implementation takes place [11]. Evaluating such implementation efforts requires answering 3 complementary questions: how was the tool introduced and perceived by its users, to what extent was it adopted and used in practice, and what was its downstream effect on daily clinical routines? This study investigates paramedics’ experiences with the implementation of the STA in the prehospital practice by evaluating the implementation process, the implementation outcomes, and its impact on clinical workflows while considering contextual factors that may have influenced these dimensions. By integrating quantitative and qualitative data, we aim to generate actionable insights for optimizing the implementation of digital health tools in prehospital emergency care.

## Methods

### Study Setting

The study was conducted within 2 emergency medical service (EMS) regions in the Netherlands: Rotterdam-Rijnmond and Zuid-Holland Zuid. This region consists of 2 EVT-capable hospitals and 7 general hospitals, where only IVT is available. Together, these EMS organizations cover a population of approximately 1.7 million inhabitants in an area of 863 km^2^ (Rotterdam-Rijnmond) and 720 km^2^ (Zuid-Holland Zuid), with a total estimated 5000 suspected strokes yearly [11,14]. Approximately 250 licensed ambulance paramedics were employed across these 2 regions. The STA was implemented in January 2024 in both regions as part of the Prehospital Triage of Patients With a Suspected Acute Stroke (PRESTO-2) study and formally introduced as the standard of care for prehospital stroke triage [15]. The STA is registered as a Conformité Européenne (CE)-compliant medical device and as a Medical Device Regulation (MDR) class I software (The European Database on Medical Devices [EUDAMED], unique device identification-device identifier [UDI-DI]: 08720892217905).

### Study Design

Embedded within the PRESTO-2 study, this study used a sequential explanatory mixed methods design [15,16]. The quantitative phase integrated objective dashboard analytics with survey data (Figure 1). The dashboard was used to monitor actual STA engagement throughout the implementation period by tracking longitudinal data (eg, monthly triage volumes and active user rates). These insights provided a baseline of system usage, while the survey captured paramedics’ initial perceptions of the implementation process, perceived usability, and attitudes toward long-term sustainability. Combined, these quantitative findings provided preliminary insights into potential implementation challenges, which informed the subsequent qualitative phase. This second phase consisted of semistructured interviews designed to contextualize and nuance the quantitative results by exploring the contextual factors shaping the implementation process, outcomes, and workflow impact.

**Figure 1.**
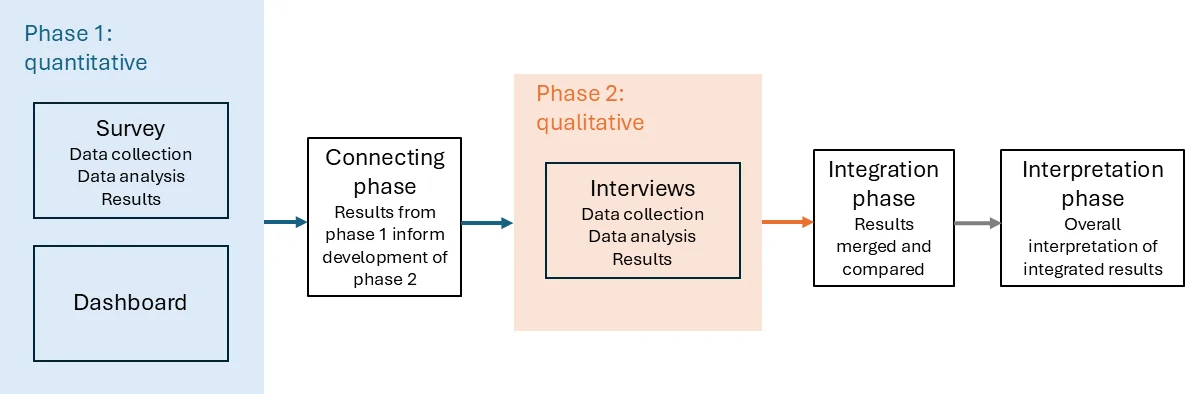
Schematic overview of the study design.

To evaluate paramedics’ experiences with the implementation of the STA, the study focused on 3 core concepts: the implementation process, the implementation outcomes, and the impact on clinical workflow (Table 1). These concepts were informed by established implementation science frameworks, including the Non-adoption, Abandonment, Scale-up, Spread, Sustainability (NASSS) Framework [3], Consolidated Framework for Implementation Research (CFIR) [17], and the Unified Theory of Acceptance and Use of Technology (UTAUT) [18] (Multimedia Appendix 1), which guided the operationalization of both the survey items and the interview guide. Methodological rigor was ensured by adhering to the Good Reporting of a Mixed Methods Study (GRAMMS) checklist [19], which provides a structured framework for transparent and comprehensive reporting of mixed methods research (Checklist 1).

**Table 1.** Operationalization of implementation dimensions.

Dimension	Definition	Key elements explored
Implementation process	The execution of the introduction strategy and how it was perceived by the users	Training and instruction, perceived technical support, promotion, and organizational communication
Implementation outcomes	The measurable results and future potential of the implementation effort	Actual usage rates (dashboard data), system usability, and perspectives on sustainability
Impact on workflow	The perceived effect of the STA^a^ on paramedics’ daily professional practice and routines	Integration into prehospital clinical workflows, perceived system usability

a STA: Stroke Triage App.

### Implementation Strategy

The introduction of the STA took place through intranet announcements, e-mail, and local ambassadors, both prior to and during the initial implementation phase. Paramedics were required to complete an online e-learning module, consisting of an instructional video and text on the STA website, as a prerequisite for account activation and clinical use. Implementation was further supported by promotional activities, including monthly newsletters, incentives (eg, cake or gift cards for every 100th patient triaged), and regular visits to ambulance stations, alongside branded promotional materials (eg, branded candy jars) to enhance visibility. Local ambassadors were appointed in each ambulance region to provide support, promote the app among colleagues, and to redirect questions to the research team. In addition, a user council consisting of paramedics was regularly consulted before and during the implementation to provide practical input on development and operational aspects. Throughout the implementation process, the use of the STA was communicated as part of the standard protocol. Although nonuse did not result in formal consequences, medical managers and the research team encouraged its use. However, use of the STA remained entirely voluntary, and paramedics were never obligated to use the app.

### Study Sample

The study population consisted of all licensed paramedics employed within the participating EMS regions. For the dashboard analysis, all active STA users during the implementation period were included. Eligibility for participation in the survey and interviews was restricted to paramedics active in clinical practice, regardless of their frequency of STA use.

### Quantitative Phase: Dashboard Analytics and Digital Survey

#### Dashboard Analytics

Objective STA usage parameters were obtained from real-time longitudinal dashboard data for both ambulance regions from the start of STA implementation until the closing of the survey (January 22, 2024, until June 17, 2025). This included the number of active accounts, the number of unique users per month, monthly triages, and number of STA usage per paramedic.

#### Digital Survey

A custom digital survey was developed by FK and EH. The survey’s functionality and content were tested in iterative rounds in which feedback was obtained from this project group, as well as the PRESTO-2 Advisory Board (consisting of regional EMS managers, paramedics, implementation researchers, and the STA software developer). Additional input was gathered during meetings within a research group focused on stroke research [20]. These review rounds led to refinements in the survey interface, item wording, and inclusion of context-specific content. The final Dutch formulated survey was hosted in Qualtrics survey software (version XM; Qualitrics LLC) [21].

The survey consisted of 39 validated as well as self-developed questions, using 5-point Likert scales (strongly agree to strongly disagree), multiple-choice questions, and open-ended fields for textual feedback (Multimedia Appendix 2). A detailed overview of the survey constructs, item counts, and psychometric properties is available (Multimedia Appendix 3). A key component of the survey was the validated Dutch version of the Digital System Usability Scale (D-SUS), which was used to assess the technical usability of the STA with a score ranging from 0 to 100, indicating poor usability for scores below 50, moderate usability for scores between 50 and 68, and good usability for scores above 68 [22]. Additionally, the survey collected demographic data, including sex, age, employment status, education level, and professional experience. The survey was filled in anonymously, and participants were invited at the end to provide contact details for follow-up interviews.

From March 27 to June 17, 2025, all licensed ambulance paramedics employed across the 2 study regions (~250 paramedics) were invited to participate. Recruitment was conducted through a multichannel approach. The survey was initially distributed by regional medical managers via e-mail, using their complete staff mailing lists, followed by a notice posted on the regional intranet. Subsequently, FK, EH, and NdR visited 3 ambulance stations per participating region to further promote the study, distributing flyers containing a QR code linking directly to the survey. A total of 2 push notifications inviting participation were additionally issued within the STA.

### Qualitative Phase: Semistructured Interviews

The interview guide was grounded in the study’s core concepts (Table 1) and further informed by survey results. Iterative revisions within the project group led to further specification of topics and rephrasing of questions. The interview guide was pilot tested between EH and NdR to evaluate the completeness and clarity of the questions, resulting in the final version (Multimedia Appendix 4).

Participants were recruited via voluntary self-selection, following an open invitation through newsletters and the on-site visits described above, in addition to an invitation at the end of the digital survey. Snowball sampling was additionally used by asking interview participants at the end of each interview to suggest colleagues with potentially different perspectives [23], though it yielded no additional participants. Data collection continued until thematic saturation was reached, assessed through interim analysis and team discussions, after which subsequent interviews yielded no new insights.

Interviews were conducted between July 8 and September 1, 2025, by either FK or EH as the primary interviewer, with NdR present in a supporting and moderating role. MS, a trained qualitative interviewer, was present during the first 4 interviews to provide support. The interviews were conducted online in Microsoft Teams, held in Dutch, and audio-recorded, pseudonymized, and transcribed verbatim in Microsoft Word by FK, EH, NdR, and RvdW. FK, NdR, and RvdW hold a medical background (MD) and were involved in the primary PRESTO-2 clinical validation study. EH and MS have a background in implementation science and were not involved in the primary clinical trial. Interviews lasted on average 39.6 (SD 6.7; range 30.6-51.6) minutes.

### Data Analysis and Integration

Survey data and dashboard metrics were analyzed descriptively using R Studio (Posit, PBC; version 4.4.2) [24] to summarize participant characteristics, system usability (D-SUS scores), and actual usage patterns. If data were distributed normally, means and SDs were calculated. Otherwise, medians with IQR values were derived. Open-text survey responses were analyzed thematically to identify recurring topics. For cross-analysis between subgroups, such as years of experience or attitude toward technology, the Pearson chi-square test was used to assess statistical significance.

Interview transcripts were analyzed in ATLAS.ti (version 24; ATLAS.ti Scientific Software Development GmbH) using an inductive approach to thematic analysis, consisting of the following phases: familiarization with the data, generation of initial codes, searching for themes, reviewing themes, defining and naming themes, and producing the report [25,26]. Codes were derived directly from the data, while the study’s 3 core concepts (Table 1) served as a sensitizing framework informing theme development. The first 2 interviews were independently coded by EH and MS. Discrepancies were discussed until consensus was reached to calibrate the coding approach, after which EH coded the remaining transcripts. Emerging themes and subthemes were iteratively reviewed within the project group, with MS providing additional verification of the interpretations.

Data integration was performed by FK and EH. Findings from the quantitative and qualitative phases were integrated to identify convergence (confirmation), complementarity (expansion), or dissonance (divergence) between the data sources. This process facilitated the development of integrated insights from both methods. The visualization of this integration is presented in a joint display, aligning the quantitative metrics with corresponding qualitative themes.

### Ethical Considerations

This study was approved by the Medical Ethics Review Committee of Erasmus Medical Center, Rotterdam, the Netherlands (MEC-2024‐0760). Informed consent was obtained electronically prior to completion. Respondents who expressed interest in follow-up interviews provided their contact details in a separate, unlinked section of the survey, and written informed consent was obtained prior to the interviews. Survey participants did not receive compensation. Interview participants each received a coffee voucher worth €10 (US $11.19) as a token of appreciation for their time. Survey data were anonymized, and interview data were pseudonymized. All data were handled in compliance with Dutch data protection regulations.

## Results

### Study Population

A total of 2075 patients were triaged using the STA before the survey ended (1679 in ambulance region Rotterdam-Rijnmond and 396 in region Zuid-Holland Zuid), corresponding with approximately 28% (2075/7500) of the expected 7500 suspected strokes within the study period of 18 months. During this period, 1855 patients were advised to go to the nearest general hospital. In 220 (11%) cases, the STA advised bypassing the general hospital and transporting the patient directly to an EVT-capable center. In 1448 (70%) cases, paramedics mentioned that they followed the advice. STA advice was not followed in 87 (4%) cases. In 540 (26%) cases, paramedics did not mention whether the STA advice was followed, since this question in the STA was not mandatory.

On June 17, 2025, 246 of the ±250 employed paramedics had an STA account. In total, 85 (±34%) paramedics responded to the survey, of which 76 consented to participate, 24 agreed to being approached for an interview, and 9 ultimately participated in the semistructured interviews (Figure 2). A total of 7 (78%) participants that participated in the semistructured interviews were male, and the mean age was 39 (SD 7) years. Overall, 4 (44%) paramedics worked full-time, and median years of experience was 6 (IQR 6‐8). Survey demographics showed that most paramedics held a neutral to positive attitude toward technology and considered themselves average or advanced in their technology experience (Table 2).

**Figure 2.**
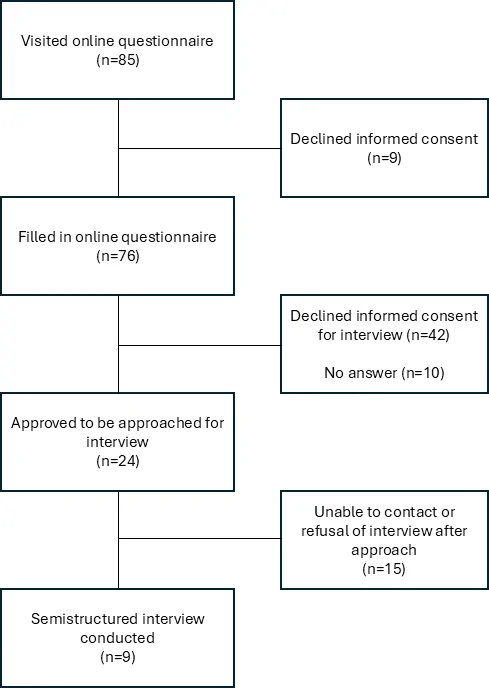
Flowchart of participant flow through the questionnaire and interviews.

**Table 2.** Baseline characteristics of participants.

Characteristics	Survey participants (n=76)
Male, n (%)	30 (46)
Age (years), mean (SD)	43 (8)
Education level, n (%)	
Senior vocational education	27 (36)
Higher professional education	31 (41)
University	2 (3)
Doctorate (PhD)	0
Other	5 (7)
Full-time (≥36 hours/week), n (%)	26 (34)
Years of experience, median (IQR)	6 (4-14)
Device used for STA^a^, n (%)	
Smartphone	35 (46)
Tablet	34 (45)
No answer	7 (9)
Attitude toward health technology, n (%)	
Very positive	11 (15)
Positive	32 (42)
Neutral	18 (24)
Negative	4 (5)
Very negative	0 (0)
No answer	11 (15)
Experience level with health technology, n (%)	
Beginner	2 (3)
Average	37 (49)
Advanced	23 (30)
Expert	3 (4)
No answer	11 (15)
Use of technology in daily life, n (%)	
Daily	63 (83)
Regular (3‐5 times/week)	2 (3)
Sometimes (1‐2 times/week)	0 (0)
Rarely (<1 time/week)	0 (0)
No answer	11 (15)

a STA: Stroke Triage App.

### Main Results

#### Overview

To investigate paramedics’ experiences with the implementation of the STA in prehospital practice, this study evaluated the implementation process, the implementation outcomes, and the tool’s impact on clinical workflows, while considering contextual factors that may have influenced these dimensions. The concurrent analysis of both quantitative and qualitative data identified 6 key themes that structure the presentation of the results. These themes are (1) onboarding and engagement, (2) usability and practicality, (3) adoption attitudes, (4) actual usage, (5) assessment and handover, and (6) future perspectives. Findings are discussed according to these themes, integrating insights from both the quantitative survey and qualitative interviews (Table 3).

**Table 3.** Integration of quantitative and qualitative findings and the mixed methods meta-inference.

Theme	Quantitative results	Qualitative results	Integrated findings
Onboarding and engagement			
Introduction	Most paramedics heard of the STA^a^ through their management (24/76, 32%), a colleague (14/76, 18%), training (11/76, 14%), or a combination of the above (15/76, 20%).	Many recalled multiple channels including e-mail, personal messages and intranet. The introduction was positively received. Early informal communication occasionally led to misconceptions.	Confirmation: multichannel exposure increases visibility of health device introduction. Expansion: clear, uniform message important.
Training	57% (43/76) mentioned receiving minimal training; 17% (13/76) did not remember any training. 41% (31/76) found training relevant; most were neutral regarding training (36/76, 47%).	The flexibility of digital modules was appreciated, in addition to practicing with test cases. The training provided was considered minimal but sufficient. However, some paramedics expressed a preference for in-person instruction and more in-depth information.	Confirmation: digital health devices training tools must be accessible, concise, yet informative. Expansion: In-person versus digital training preference varies per participant.
Promotion	Most paramedics found promotion somewhat helpful (25/76, 33%). 13% (10/76) found it very helpful. Promotion was widely visible.	Promotion was seen by all interviewed paramedics and was overall deemed as helpful for visibility. Feedback, case discussions, and acknowledgment of users’ efforts were considered essential for maintaining awareness and reinforcing involvement.	Confirmation: Repetitive promotion, timely feedback from support team, and acknowledgment of efforts are essential for visibility of digital health tool and implementation.
Usability and practicality			
Usability and practicality	Median D-SUS^b^ score was 67.5 (IQR 57.50‐77.50), corresponding with average usability, with a range of 35 to 92.5. 79% (60/76) found the STA easy or very easy to understand. 63% (48/76) found the STA accurate or very accurate.	The app was perceived as easy and intuitive to use. Its use in acute situations was sometimes challenging, as immediate patient care took priority. The tool was reported to be less suitable for patients with atypical or subtle symptoms.	Confirmation: Ease of use and understandability is important. Minimization of technical barriers for app usage is crucial, such as login or mobile compatibility. Contradiction: Even with ease of use, there may still be technical and clinical barriers in urgent settings that decrease digital health tool usability.
Adoption attitudes			
Demotivating and motivating factors	69% (52/76) had no or very few fears regarding STA use. 16% (12/76) were neutral; 1% (1/76) did have fears regarding STA use. Overall trust in the STA was high.	Confidence in the tool increased with repeated use. Limited understanding of the rationale underlying STA advice sometimes led to frustration and reduced confidence, which could be restored after consultation from the research team.	Confirmation: No fear regarding usage of STA. Transparency and explanation with the given advice is crucial for confidence.
Actual usage	2075 triages were performed until survey completion. The total number of monthly triages ranged from 52 to 153 (IQR 107‐131). 246 active accounts were registered. Frequency of STA use ranged from 1 to 47, median 7 times (IQR 3‐13). Unique users per month ranged from 40 to 94, median 81 (IQR 74‐83). 96% (73/76) had used the STA at the time of survey completion. Median duration of use was 12 months (IQR 10 to 12; range 3‐14). In the month prior to the survey, 40% (30/76) used the STA in more than 60% of eligible cases, 20% (15/76) in 40% to 60%, 13% (10/76) in 20% to 40%, and 17% (13/76) in less than 20%. Regarding completion, 33 paramedics (43%) reported that they often completed all STA steps, 27 (36%) indicated that they always did so. STA use remained stable for 43 respondents (57%), increased for 11 (15%), and decreased for 15 (20%). Smartphones (35/76, 46%) and tablets (34/76, 45%) were used almost equally to access the application.	During early phases of implementation, app usage was not yet routine practice. It was sometimes unclear whether STA use was standard protocol or optional for research purposes. Paramedics were less likely to use the STA when an LVO^c^ was clinically apparent or when they considered their own clinical judgment sufficient.	Expansion: STA usage varies greatly among paramedics, as well as varying in time. These factors are dependent on the integration of workflow routine, practical and technical barriers, and perceived added value.
Assessment and handover	Median reported STA use was 5 minutes (IQR 3‐5). 73 participants used the STA (73/76, 96%). 57% (43/76) of paramedics’ use has stayed equal during STA deployment. 15% (11/76) reported increased STA use; 20% (15/76) reported decreased STA use. Most often, they completed or always completed all STA steps (60/76, 79%). 34% (25/76) found their workflow improved or improved greatly, 47% (36/76) found their workflow stayed the same. 5% (4/76) found their workflow had gotten worse.	Interview respondents generally perceived the STA as a small component within their broader tasks of responsibilities, which did not substantially alter their workflow. STA provided support in communication with neurologists, reducing discussion time in accordance with regional care agreements, and paramedics appreciated the systematic overview of the patient. Paramedics felt that the STA use contributed to improved patient outcomes and fewer interhospital transfers.	Confirmation: The STA had minimal impact on clinical workflow. Expansion: The STA may save time through more effective communication, less discussion, and a more systematic neurological examination, leading to better clinical understanding.
Future perspectives	59% (45/76) of paramedics were likely or very likely to use the STA in the future. 17% (13/76) were neutral, 8% (6/76) were not likely to use the STA in the future.	Continued use was considered acceptable only if the tool provides clear added clinical value and is easy to use. For future implementation, paramedics emphasized the importance of improved usability and workflow integration.	Confirmation: Positive intentions for future use, but is conditional on added clinical value, workflow integration, and technical ease of use.

a STA: Stroke Triage App.

b D-SUS: Digital System Usability Scale.

c LVO: large vessel occlusion

#### Theme 1: Onboarding and Engagement

Most respondents reported in the survey that they first learned about the STA through their management (24/76, 32%). In the interviews, however, several respondents described learning about the STA through multiple channels, including e-mail, personal messages, and the intranet. Survey respondents perceived the promotion of the STA generally as helpful: 25 (33%) rated it as somewhat helpful, 22 (29%) were neutral, and 10 (13%) found it very positive. Interviewees generally spoke positively about the introduction, noting that repeated promotion (including newsletters, dashboard access, posters, on-site visits, and rapid responses from the research team) helped them remain engaged with the STA.

In the beginning, the app sparked a lot of conversation among colleagues. However, as it was used for a longer period, it became less special and everyone developed their own way of working with it. The promotion was very well done and showed that the research team genuinely put in effort. Still, over time, we found our own routine with STA, and promotion became less impactful once everyone was already familiar.

[P4]

Early informal communication during the implementation process occasionally caused misunderstandings. For example, some interviewees reported fears that using the STA would be mandatory or would reduce their autonomy. Respondents described that clear and timely communication between regional medical managers, research nurses, and the research team helped prevent these misunderstandings and increased their acceptance of the STA.

It felt like we had to fill in something for every neurological patient, which made me worry about losing autonomy and turning exams into a rigid step-by-step process.

[P8]

Regarding training, survey respondents reported varying experiences. A total of 43 (57%) respondents reported following minimal training, while 13 (17%) did not remember receiving any training. Most respondents found the training relevant (31/76, 41%) and 24 (32%) considered it efficient, whereas 36 (47%) were neutral. Interviewees appreciated the flexibility of digital modules and the opportunity to practice with test cases, although some mentioned that they would have preferred in-person training for a deeper practical understanding.

'I found the training clear. Everything was fine. It was clear what needed to be scored and how. So technologically, it was straightforward.

[P3]

I remember completing the training, but I felt the background information could have been more comprehensive; it was minimal. I would have liked to understand why we needed to perform the tests within the RACE score.

[P4]

Interviewees emphasized that feedback, case discussions, and acknowledgment of their efforts through small tokens of appreciation (eg, candy or gift cards) were essential for maintaining awareness of the STA and reinforcing engagement. Respondents noted that their interest tended to fade once the initial novelty wore off. Case-based learning and real-world examples further motivated paramedics by demonstrating the STA’s added clinical value. Interviewees explained that local ambassadors at ambulance posts supported colleagues and encouraged uptake, but highlighted that familiarization took time, and periods without stroke cases sometimes led to forgetting the STA. Some paramedics admitted postponing practice with the STA despite encouragement, which occasionally resulted in difficulties during real encounters.

In the early phase, colleagues could open the app, but no one was really using it yet. It took a little time before people thought, ‘Oh right, let’s fill in the app.’

[P4]

#### Theme 2: Usability and Practicality

Survey respondents generally reported that the STA was easy to understand: 37 paramedics (49%) rated it as easy, and 23 ( 30%) rated it as very easy. Regarding accuracy, 41 respondents (54%) considered the STA advice accurate, with 7 (9%) rating it as very accurate. These survey findings were supported by interviewees, who described the STA as intuitive, quick, and straightforward to use, highlighting its practical value in everyday clinical situations.

And when I tried it once during a slow shift, it worked fine, so I didn’t have any problems with it.

[P1]

However, interviewees also emphasized that STA use in acute situations could be challenging, as immediate patient care took priority. They described practical and technical barriers, including short transport times, hygiene constraints, multitasking, login difficulties, and complex patient presentations, that sometimes led to delayed or postponed STA use after initial treatment. Several respondents noted that the tool was less suitable for patients with atypical or subtle symptoms. In some cases, these barriers resulted in nonuse altogether, for instance in unstable patients, very short transport times, or proximity to an intervention center.

It’s an extra step, which usually isn’t a problem, but we typically want to do it once we’re in the ambulance and on the way to the hospital, that’s when we handle the paperwork. If it’s a suspected stroke, do we really want to open the app before leaving to decide where to go? It adds time, especially if you must find your password, and it’s not ideal when the patient has vomited, a head wound, or any other urgent issue. That’s not the best moment to pull out your phone and look for a password.

[P1]

The times I don’t use the app are usually when I already know it will be a general hospital, or the situation is difficult: it’s dark, which makes assessing the patient difficult, the patient is vomiting, or the communication with the family is challenging. That makes it extra hard. Most of the time, it ends up being a general hospital anyway. When I do fill it in, it usually doesn’t surprise me.

[P3]

The median D-SUS score was 67.5 (IQR 57.5‐77.5; range 35‐92.5), indicating average usability. Individual item scores (Figure 3) showed that survey respondents generally found the STA easy to use and felt able to learn how to operate the tool quickly. No statistically significant difference was found between D-SUS scores and attitude toward technology (*P*=.11) and D-SUS scores and years of experience (*P*=.36).

**Figure 3.**
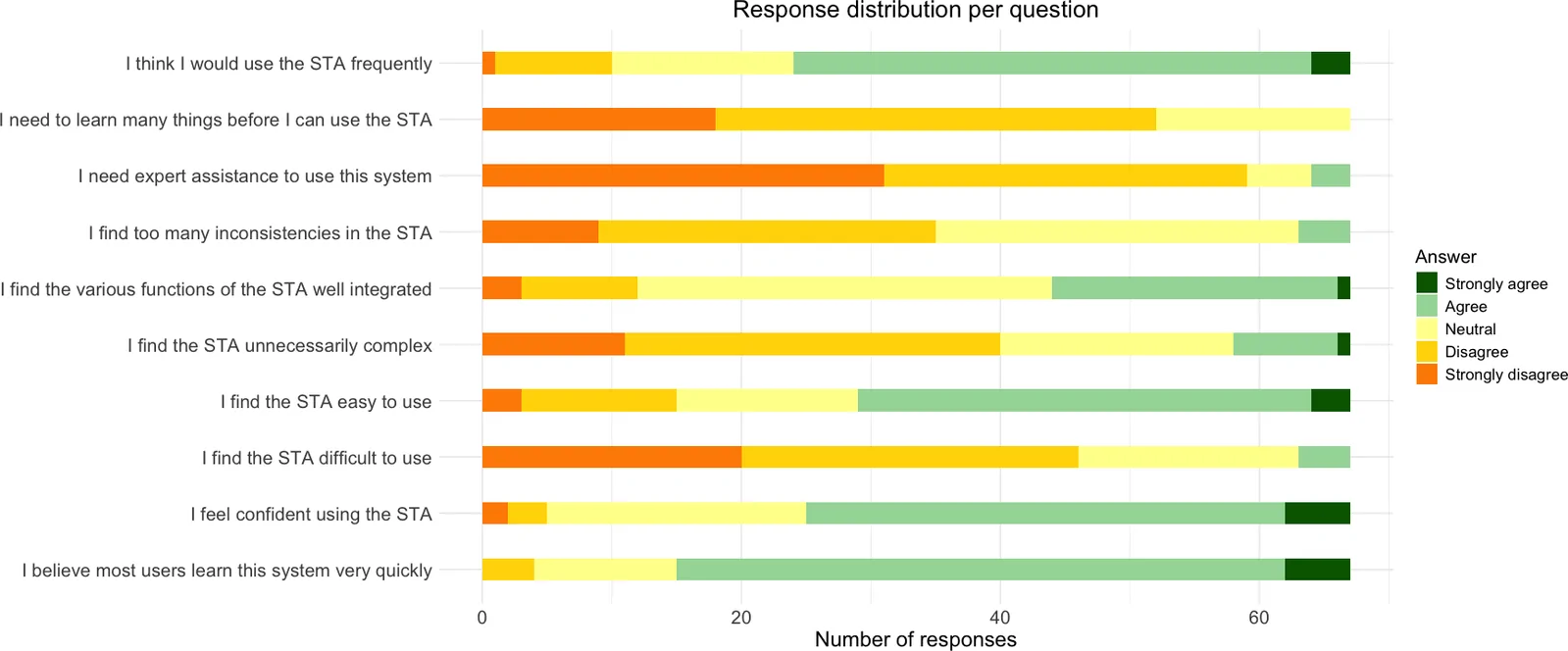
Overview of Digital System Usability Scale scores per item. STA: Stroke Triage App.

#### Theme 3: Adoption Attitudes

Survey respondents reported generally low levels of fear when using the STA: 33 (44%) respondents indicated no fear, while 19 (25%) respondents reported only minor concerns. In contrast, 12 respondents were neutral (16%), and 1 (1%) reported to be very worried. Interviewees similarly described a high level of trust in the STA. They explained that confidence in the tool increased with repeated use, particularly when its recommendations aligned with their own clinical judgment, which strengthened their understanding of the STA’s predictions. Interviewees also noted that the scientific foundation of the STA and the involvement of the research team further contributed to their trust.

However, interviewees reported that limited understanding of the rationale and the contextual factors underlying the STA’s recommendations sometimes led to uncertainty and frustration, particularly when the advice conflicted with their intuitive clinical judgment, occasionally resulting in reduced confidence and decreased use. They indicated that consultation with the research team and clarification of the advice generally helped to restore their confidence.

You also get answers very quickly when you ask questions, which makes you notice, even on a shift, that the study group is involved and that your concerns are heard. For example, if a case didn’t go perfectly with another hospital, you can find out exactly what happened and where things went wrong.

[P8]

At the same time, interviewees emphasized that the STA was still often associated with a research context, leading some respondents to perceive it as optional rather than as part of routine care. More experienced users sometimes viewed the STA as an additional step rather than a clear benefit, making them less likely to use the app when an LVO was clinically apparent or when they considered their own clinical judgment sufficient. In contrast, others reported being more inclined to use the STA when they suspected an LVO, as it enabled direct bypass to an intervention center without the need for further discussion during handover.

Overall, interviewees regarded the STA as a supportive tool that enhanced, rather than replaced, their clinical decision-making.

#### Theme 4: Actual Usage

Dashboard data showed that the monthly number of STA triages ranged from 52 to 153, with a median of 120 (IQR 107‐131; Figure 4). Individual STA use ranged from 1 to 47 times, with a median of 7 (IQR 3‐13). The number of monthly unique users varied between 40 to 94, with a median of 81 (IQR 74‐83).

Most survey respondents reported having used the STA at the time of the survey (73/76, 96%), with a median duration of use of 12 months (IQR 10‐12). In terms of frequency, 30 (40%) respondents reported using the STA in more than 60% of eligible cases, whereas only 13 participants used it in less than 20% (n=13, 17%) of cases. Regarding completion, most paramedics indicated that they often (n=33, 43%) or always (n=27, 36%) completed all required triage steps. For the majority, STA use remained stable (n=43, 57%) or increased over time (n=11, 15%). To access the app in the field, paramedics were almost equally divided between the use of smartphones (n=35, 46%) and tablets (n=34, 45%)

**Figure 4.**
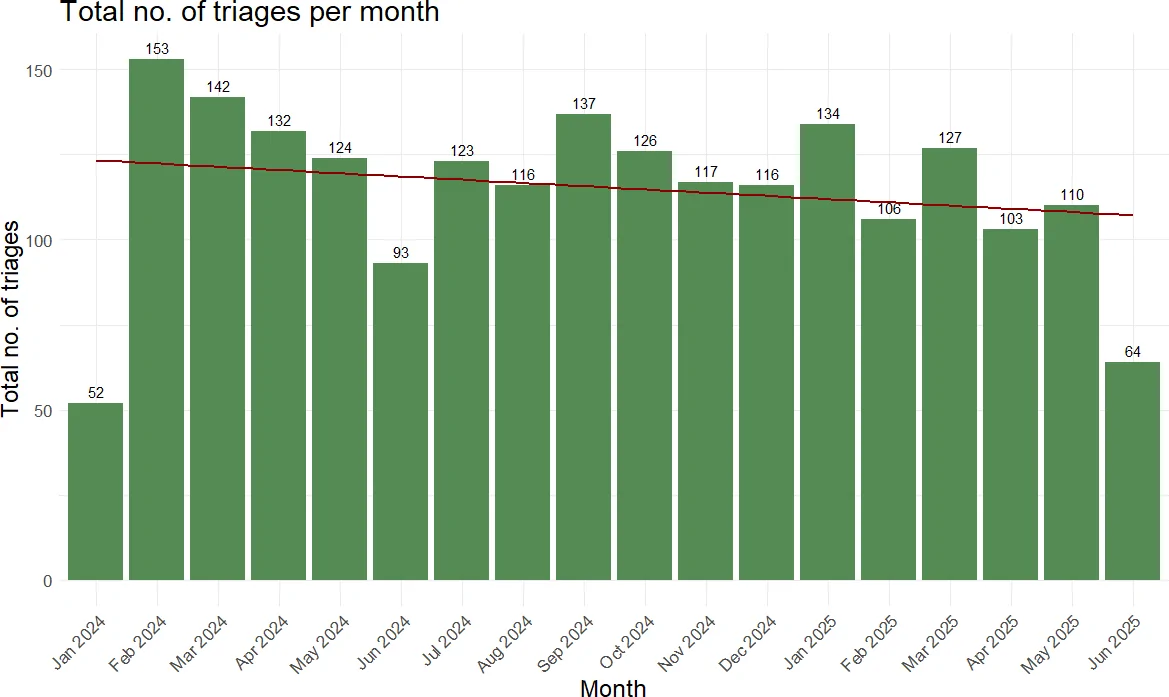
Overview of number of Stroke Triage App triages per month from January 2024 until end of survey collection in June 2025.

Interviewees provided additional context to these usage patterns. They mentioned that they sometimes forgot to use the app in acute situations, primarily because it was not yet routine, patients were unstable, technical issues arose, or due to partial postponement of use. Additionally, for some paramedics it was initially unclear whether STA use was standard protocol or optional for research purposes.

I have to admit, it took me a while to use the app for the first time, I kept forgetting. For the first 10 thrombolysis cases, I didn’t use it at all. It wasn’t on my radar yet, and I went on autopilot straight to the nearest hospital. Afterwards I thought, ‘Oh right, I should have filled it in,’ but by then it didn’t really make sense because I was already there. Now it’s part of my routine, but it took some time to get there.

[P6]

Interviewees mentioned that reminders from neurologists at the emergency department helped reinforce routine use of the STA. They described that use was more likely in lower-urgency situations, where there was sufficient time for decision-making.

#### Theme 5: Assessment and Handover

Survey respondents reported mixed perceptions regarding the impact of the STA on their workflow: 23 (30%) respondents indicated that the STA improved their workflow, 2 (3%) reported a substantial improvement. In contrast, 36 (47%) respondents reported no impact, and 4 (5%) reported a worsened workflow. Interviewees generally described the STA as a small component within their broader tasks of responsibilities, which did not substantially alter their workflow. However, they noted that the STA increased their awareness of patient urgency, particularly in cases involving large neurological deficits, and supported the identification of LVOs. Interviewees further explained that the STA supported neurological assessment by providing a structured approach to clinical evaluation. Interviewees reported that the tool also facilitated communication with neurologists by streamlining handovers (eg, through clear reporting of RACE scores) and reducing the need for discussion, thereby minimizing time lost in the acute care phase. In addition, interviewees valued the clear overview of patients’ neurological status provided by the STA. Overall, interviewees felt that STA use contributed to improved patient outcomes and fewer interhospital transfers.

… the fact that the app makes the decision for you by giving the advice, it really helps. You don’t have to decide yourself if you’re unsure, which makes it easier. Filling in the app gives you an immediate overview of the patient (especially since the RACE score can sometimes be tricky) and clearly shows where to go. I think that reduces a lot of uncertainty. In addition, the app helps with the neurological assessment itself, allowing you to hand over your findings more effectively to the neurologist.

[P2]

#### Theme 6: Future Perspectives

Most survey respondents indicated that they were likely (26/76, 34%), or very likely (19/76, 25%) to use the STA in the future. No statistically significant effect was found between years of experience and the likelihood to use the STA in the future (*P*=.68), and attitude toward technology and the likelihood to use the STA in the future (*P*=.38). Interviewees emphasized that continued use would only be acceptable if the tool provided clear added clinical value and remained easy to use. Several interviewees suggested a more selective application of the STA, noting that its recommendations often aligned with their own clinical judgment, which made its added value difficult to assess. They also perceived the STA as less useful for patients with a low likelihood of stroke or in cases with an obvious LVO, where destination decisions were already clear. For example, when the nearest hospital was already an EVT-capable center, interviewees reported no added clinical value of the STA, except for clear communication during handover.

For future implementation, interviewees highlighted the importance of improved usability and workflow integration. Suggested improvements included minimizing the number of required actions, simplifying or eliminating login procedures, integrating the STA with the electronic ambulance ride form to avoid duplicate data entry, and implementing a feedback loop with hospital diagnoses to validate paramedics’ triage decisions. Finally, participants mentioned that enabling automatic transfer of information (eg, RACE scores) to hospitals would be recommended. Enhancing mobile compatibility was also recommended for ease of use.

It needs to be practical, easy to use, and genuinely add value. So, if your conclusion is that it does provide real benefits, the next step should be to make the app as accessible as possible. If the added value is limited, we should seriously reconsider whether it’s worth continuing.

[P1]

In addition, interviewees expressed a clear preference for brief explanations underlying the STA’s recommendations, which they felt would support understanding and legitimize decision-making, particularly when the advice diverged from their clinical intuition. They emphasized that sustained implementation would depend on consistent communication, clarifying that the STA is not optional, and maintaining realistic expectations regarding paramedics’ roles and responsibilities in acute stroke care.

## Discussion

### Principal Findings

This study aimed to investigate paramedics’ experiences with the implementation of the STA in real-world prehospital practice by evaluating the implementation process, the implementation outcomes, and its impact on clinical workflows, while considering contextual factors that may have influenced these dimensions. Despite the identified barriers in usability and clinical added value, the STA achieved substantial initial reach supported by a structured implementation strategy. However, the high self-reported acceptability in the survey did not automatically translate into consistent, routine integration into daily prehospital workflows. Instead, actual usage varied with practical and contextual factors and paramedics’ perceived added value. While the STA facilitated structured neurological assessments and enhanced communication during handovers, it simultaneously induced workflow friction for experienced users who viewed the app as an additional step in high-stakes environments.

Dashboard data indicated a decline in usage after an initial peak, reflecting the “novelty fade” phenomenon in digital health implementation [27]. This suggests that initial engagement was driven more by novelty than by structural integration. Qualitative insights indicate that the low frequency of stroke cases during shifts formed a structural barrier, consistent with reports that fewer than 5% of ambulance rides involve suspected stroke cases [28]. Literature on habit formation suggests that this lack of exposure limits paramedics from moving past the initial learning phase to an automated workflow integration before the novelty fades [29,30]. Moreover, trust in clinical decision support systems is typically built through consistent positive reinforcement over time [31]. In a low-frequency setting, a technical barrier or ambiguous clinical results may therefore carry disproportionate weight, undermining perceived reliability and long-term use [31]. This aligns with comparable findings on the operational burden of screen-based tools in time-critical prehospital settings, where balancing digital support with the demands of acute care has similarly been identified as a barrier [32]. This concern was further reinforced by the misconception that the STA was a temporary research tool, rather than standard care, creating a sense of perceived optionality, a known barrier to long-term adoption [33]. Together, these factors point to a structural tension in acute-care digital health implementation, in which reinforcement needed to build trust and routine use is inherently difficult to achieve in a low-frequency clinical context.

The findings of this study reflect a common, yet complex trajectory in digital health implementation, where high levels of acceptability and perceived appropriateness do not automatically ensure long-term sustainability [34]. However, the emergence of a core group of persistent users, the early adopters, indicates that the STA has reached a level of routinization for a subset of the population [35]. Yet, the observed decline in usage suggests that the tipping point for the early majority has not yet been fully reached [35], as members of this group may have been more sensitive to the structural barriers identified in this study. Our findings also point to a pattern of selective, strategic use across users, where the STA is reserved for cases of clinical uncertainty rather than applied universally. Rather than viewing this as a lack of adherence, this selective approach aligns with the concept of professional autonomy in high-stakes environments, where clinical decision-support tools are reserved for complex decision-making scenarios [36]. However, this approach assumes that paramedics can accurately identify a case as “unmistakable” before deciding to bypass the STA. Such judgments may be less reliable in atypical presentations, where the STA is likely to provide the greatest added value. This tension is not unique to the STA: in the ParaNASPP study [2], which evaluated a comparable prehospital stroke-recognition tool, paramedics similarly reported relying on their own clinical judgment when classic stroke symptoms were clearly present, while placing greater reliance on the tool in more ambiguous presentations.

### Practical Implications

To ensure long-term sustainability of the STA, several practical implications must be addressed. First, technical complexity should be minimized by embedding the STA directly within existing prehospital information systems, to eliminate repetitive login procedures and facilitate automatic data transfers to receiving hospitals. More broadly, this illustrates that decision-support tools are more likely to be sustained when they are embedded within existing clinical systems rather than operating as stand-alone apps. Second, the clinical relevance of the STA can be strengthened by establishing a feedback loop that provides paramedics with formal confirmation of hospital diagnoses, addressing the need for paramedics to validate both the app’s recommendations and their own clinical triage decisions against actual outcomes in order to calibrate their trust in the tool. Increasing the transparency of the STA by providing the clinical rationale underlying its recommendations could help mitigate the “black box” effect, particularly when in situations where the app’s advice differs from a paramedic’s initial judgment. Lastly, given the low frequency of stroke cases per shift, sustained promotion of the tool remains essential. Future implementation efforts should explicitly frame the STA as a specialized resource for cases of diagnostic uncertainty rather than a replacement for professional judgment. This may be particularly important, as infrequent exposure to decision-support tools may limit opportunities for paramedics to develop a clear understanding of their intended role and limitations.

### Future Recommendations

Future research should prioritize longitudinal studies to evaluate the effectiveness of diverse feedback mechanisms, such as providing paramedics with real-time data on hospital diagnoses and more detailed insights into the underlying clinical rationale of the STA advice. Such feedback is essential to determine whether it enhances the perceived relative advantage and long-term adherence to the tool. Furthermore, since this study was primarily conducted in an urban setting with high hospital density, future research should explore whether the utility of the STA is more evident in rural areas, where longer transport times and fewer hospital options might lead to a larger impact on treatment times. Finally, further investigation into the receiving end of the acute stroke chain is needed to assess how neurologists perceive and use the STA data during the handover. Exploring potential changes in inter-professional dynamics and the perceived clinical authority of app-generated recommendations is needed for optimizing the integration of the STA into the broader acute care pathway.

### Strengths and Limitations

A key strength of this study is the evaluation of the STA within a real-world prehospital setting over an extended period of 1.5 years. Digital health interventions in prehospital care are frequently evaluated in testing or development settings rather than in routine clinical practice, leaving evidence on real-world implementation and sustained use relatively scarce [6,37]. The longitudinal approach in this study captured long-term usage patterns and perspectives, providing a nuanced insight into the sustainability of the implementation process. The credibility of the results is supported by the fact that 96% of survey respondents and all interview participants had actively used the STA, ensuring that the findings reflect actual clinical experience rather than hypothetical expectations. Furthermore, because the results of the primary clinical validation study were not yet available during data collection, the risk of participants altering their feedback based on the STA’s proven clinical efficacy was minimized. Moreover, the study’s grounding in multiple implementation science frameworks enabled comprehensive operationalization of both instruments. For instance, the UTAUT provided measurable items, while CFIR’s implementation process domain structured the qualitative exploration of training, communication, and support. Ultimately, this study addresses the current lack in prehospital implementation research by providing a detailed end-user perspective on prehospital implementation through a comprehensive mixed methods approach [38].

Several limitations should be considered when interpreting our findings. First, despite efforts to include a diverse range of perspectives, selection bias may have occurred, potentially overrepresenting paramedics with a positive attitude toward the STA. A consequence of the high participation rate among active users is the potential omission of critical insights from nonusers, whose reasons for nonuse remain largely unexplored. Although the survey was actively promoted with an emphasis that feedback from all viewpoints was essential, no responses were received from this subgroup, leaving the dedicated nonuser section of the survey entirely uncompleted. Similarly, while snowball sampling was used to recruit critical users for the qualitative interviews, this subgroup remained underrepresented. Consequently, our understanding of the barriers faced by those who did not use the STA remains limited. Second, the involvement of some researchers in both this study and the primary clinical validation trial could have introduced a risk of confirmation bias, potentially leading to an overinterpretation of positive findings. To mitigate this risk, researchers who were independent of the main clinical trial were involved in conducting interviews and performing the thematic analysis. This approach brought an outsider perspective to the data, enhancing the reflexivity and interpretive rigor in the analysis. Finally, the urban geographical context of the current study regions may limit the generalizability of our results to more rural settings. In areas where the intervention center was the closest hospital, the perceived urgency of the clinical decision problem was likely reduced. This proximity may have led to lower STA compliance compared to regions with longer transport times and more complex triage choices, where the relative advantage of a decision-support tool might be more apparent.

### Conclusion

This study demonstrates that the STA achieved substantial reach and initial adoption within a prehospital setting, facilitating structured neurological assessments and establishing a shared clinical language between paramedics and neurologists. However, the widespread acceptance did not consistently translate into routine, sustained integration into daily practice. Usage remained selective, influenced by paramedics’ professional judgment in situations of clinical uncertainty rather than applied universally. Beyond its immediate findings, this study contributes to the STA’s ongoing development and to broader efforts toward sustainable digital health implementation in prehospital emergency care. Ultimately, successful implementation depends as much on addressing organizational and human factors as on technical performance.

## Supplementary material

10.2196/105987 Multimedia Appendix 1 Theoretical approaches informing survey and interview guide design.

10.2196/105987 Multimedia Appendix 2 Semisemistructured interview guide on experiences with the STA, including main questions and probes (English version).

10.2196/105987 Multimedia Appendix 3 Overview of survey constructs, item characteristics, scoring, and psychometric properties.

10.2196/105987 Multimedia Appendix 4 Semistructured survey on experiences with Stroke Triage App, including all questions and response options (English version).

10.2196/105987 Checklist 1 Good Reporting of A Mixed Methods Study (GRAMMS) checklist.

## Abbreviations

CE: Conformité Européenne
CFIR: Consolidated Framework for Implementation Research
D-SUS: Digital System Usability Scale
EMS: emergency medical service
EUDAMED: The European Database on Medical Devices
EVT: endovascular treatment
GRAMMS: Good Reporting of a Mixed Methods Study
IVT: intravenous thrombolysis
LVO: large vessel occlusion
MDR: medical device regulation
NASSS: Non-adoption, Abandonment, Scale-up, Spread, Sustainability Framework
PRESTO-2: Prehospital Triage of Patients With a Suspected Acute Stroke
RACE: Rapid Arterial Occlusion Evaluation
STA: Stroke Triage App
UDI-DI: unique device identification-device identifier
UTAUT: Unified Theory of Acceptance and Use of Technology
