# Supplementary material for: Implementation and Evaluation of the Stroke Triage App Among Paramedics in Prehospital Emergency Care: Sequential Mixed Methods Study
Source: J Med Internet Res. 2026 Nov 3;28:e105987. doi: 10.2196/105987 (PMC13735210; doi:10.2196/105987)
Supplement: Supplementary file 1 [file jmir-v28-e105987-s001.docx]

Multimedia Appendix 1. Theoretical approaches informing survey and interview guide design

| Framework (full name) | Key focus | Domains/ constructs used | Operationalized in |
| --- | --- | --- | --- |
| Non-adoption, Abandonment, Scale-up, Spread, Sustainability Framework  (NASSS^1^) | Explains the complexity of technology adoption and sustainability in healthcare settings | The technology, the adopters, the organization, embedding & adaptation over time | Survey: Q2c, Q5, Q6, Q9, Q10-14, Q26 – 28, Q30 Interview: Impact on workflow, technology effort expectancy & self-efficacy, future intention to use |
| Consolidated Framework for Implementation Research  (CFIR^2^) | Assesses factors affecting implementation success | Implementation process, intervention characteristics, inner setting, characteristics of individuals | Survey: Q2a-2c, Q5, Q6, Q10-14,  Q26 –28, Q30, Q37, Q39 Interview: Implementation process, impact on workflow, actual use, app accuracy |
| Unified Theory of Acceptance and Use of Technology  (UTAUT^3^) | Examines user acceptance and use of technology | Performance expectancy, effort expectancy, social influence, anxiety, self-efficacy, behavioral intention | Survey: Q2b–c, Q15–24, Q26–29, Q31, Q37–39 Interview: Demographic characteristics section |
| Dutch System Usability Scale  (D-SUS^4^) | Measures perceived usability of digital systems | Full 10-item scale | Survey: Q15-Q24 |

References

1.	Greenhalgh T, Wherton J, Papoutsi C, et al. Beyond Adoption: A New Framework for Theorizing and Evaluating Nonadoption, Abandonment, and Challenges to the Scale-Up, Spread, and Sustainability of Health and Care Technologies. *J Med Internet Res* 2017; 19: e367. 2017/11/03.

2.	Damschroder LJ, Aron DC, Keith RE, et al. Fostering implementation of health services research findings into practice: a consolidated framework for advancing implementation science. *Implement Sci* 2009; 4: 50. 2009/08/12.

3.	Venkatesh V, Morris, M. G., Davis, G. B., & Davis, F. D. Unified Theory of Acceptance and Use of Technology (UTAUT). *APA PsycTests* 2003.

4.	Ensink CJ, Keijsers NLW and Groen BE. Translation and validation of the System Usability Scale to a Dutch version: D-SUS. *Disabil Rehabil* 2024; 46: 395-400. 2022/12/28.
