# Supplementary material for: Implementation and Evaluation of the Stroke Triage App Among Paramedics in Prehospital Emergency Care: Sequential Mixed Methods Study
Source: J Med Internet Res. 2026 Nov 3;28:e105987. doi: 10.2196/105987 (PMC13735210; doi:10.2196/105987)
Supplement: Supplementary file 2 [file jmir-v28-e105987-s002.docx]

**Multimedia Appendix 3: Semi-structured survey**

*Informed Consent for the Study on the Stroke Triage App*

*Study Title:* “Process and impact evaluation of the Stroke Triage App: a sequential mixed-methods study” *Researchers:* This study is conducted by Femke Kremers and Ellen Hu, under the supervision of Prof. Dr. Bob Roozenbeek and Dr. Sandra Sülz, on behalf of Erasmus MC and Erasmus University Rotterdam. The study has been approved by the Medical Ethics Review Committee of Erasmus MC (MEC number MEC-2024-0760). *Purpose of the Study:* This study evaluates the use and effectiveness of the Stroke Triage App in practice among ambulance nurses in the Netherlands. The Stroke Triage App has been developed to improve triage in patients with a suspected stroke. We investigate how the app is used, how its usability is experienced, and which factors influence this. *Study Method:* In this study, an electronic survey will be distributed to collect data on the use of the Stroke Triage App and its impact on clinical practice. *Participation:* Completing the survey takes approximately 10 minutes. Participation is entirely voluntary. You may stop at any time and resume later, as your responses will be saved in the meantime. *Privacy:* All responses will remain confidential. Only the researchers will have access to the data. The data will be used exclusively for this study and will not be shared with third parties. *Risks:* There are no risks associated with participation. Participants may choose not to take part in the survey. *Contact:* If you have any questions, you may contact:
Femke Kremers: f.kremers@erasmusmc.nl
Ellen Hu: hu@eshpm.eur.nl

For detailed information about the study, you may read the full information letter in the attached document.

*Informed consent*

By clicking “I agree,” you indicate that you voluntarily and informedly participate in this study. Your name and signature will be collected solely for consent purposes in accordance with the guidelines of the Medical Ethics Review Committee of Erasmus MC (MEC number MEC-2024-0760). After collection, your data will be processed in such a way that they can no longer be directly traced back to you. Your responses will be processed anonymously, and identifiable data will be stored separately.

○ I agree

○ I do not agree (-> go section *Follow-up*)

If you agree, please provide your name & signature

(open text field)

*Familiarity with and experiences using the Stroke Triage App*

In this section, we ask about your familiarity with and experience using the Stroke Triage App.

Q1 How did you first hear about the Stroke Triage App?
*(Multiple answers possible, please select all that apply)*

▢ Through a colleague

▢ Supervisor or management

▢ Training session/workshop

▢ Social media

▢ Other

Q2 Have you used the Stroke Triage App?

○ Yes
 ○ No (-> continue with Q2a – Q2d)

Q2a What is the reason you have not used the Stroke Triage App?
*(Multiple answers possible, please select all that apply)*

▢ Lack of awareness of the application

▢ Lack of training

▢ Technical issues

▢ Lack of time

▢ Lack of trust in the application

▢ Preference for existing methods

▢ There has not yet been a stroke case in which to use the app

▢ Other (open text field)

Q2b Do you know colleagues in your professional network who use the Stroke Triage App?

○ Yes, multiple colleagues
○ Yes, a few colleagues
○ No, I do not know anyone who uses the Stroke Triage App
○ I don’t know

Q2c Which factors would influence your decision to use the Stroke Triage App?
*(Multiple answers possible, please select all that apply)*

▢ More training and guidance in using the app
▢ Improved technical stability and reliability
▢ Faster access to and greater ease of use of the ap
▢ Greater confidence in the accuracy of the recommendations
▢ Better integration with existing workflows and systems
▢ Positive experiences or recommendations from colleagues
▢ More information about the benefits and effectiveness of the app
▢ Other (open text field)

Q2d To what extent are you considering using the Stroke Triage App?

○ Very likely (1)
○ Likely (2)
○ Neutral (3)
○ Unlikely (4)
○ Very unlikely (5)
○ I don’t know (6)

*Use of the Stroke Triage App*

In this section, we aim to gain insight into how you use the Stroke Triage App in your work.

Q3 How many months have you been using the Stroke Triage App?
*(Please provide your answer in full months)*

(Open text field)

Q4 How often have you used the Stroke Triage App in the past month when managing a suspected stroke?

○ More than 60%
○ Approximately 40–60%
○ Approximately 20–40%
○ Less than 20%
○ Never

Q5 How has your use of the Stroke Triage App changed since you first started using it?

○ Increased (1)
 ○ Remained the same (2)
 ○ Decreased (4)
 ○ Stopped (3)

Q6 If your use of the Stroke Triage App has changed since you first started, what was the main reason for this change?
 (Open text field)

Q7 On average, how many minutes do you spend using the Stroke Triage App each time?
*(Please provide your answer in full minutes)*	(Open text field)

Q8 How often do you complete all the steps of the full procedure in the Stroke Triage App during use?

○ Always
○ Often
○ Sometimes
○ Rarely
○ Never

Q9 On which device do you usually use the Stroke Triage App?

○ Tablet
○ Smartphone
○ Laptop
○ Other

*Training and Promotion*

In this section, we ask about the training and promotion you have received regarding the use of the Stroke Triage App.

Q10 To what extent has the promotion of the Stroke Triage App helped you to better understand the app?

○ Very helpful
○ Somewhat helpful
○ Neutral
○ Hardly helpful
○ Not helpful at all
○ I have not received any promotion

Q11 To what extent have you received training on using the Stroke Triage App?

○ Extensive training (e.g., multiple sessions, practical exercises)
○ Some training (e.g., attended one or two sessions)
○ Minimal training (e.g., a brief introduction)
○ I have not received any training

Q12 How relevant did you find the training to your daily work?

○ Very relevant
○ Relevant
○ Neutral
○ Not relevant
○ Not relevant at all

Q13 How effective did you find the training in preparing you to use the Stroke Triage App?

○ Very effective
○ Effective
○ Neutral
○ Ineffective
○ Completely ineffective

Q14 How did you experience the training and promotion related to support for the Stroke Triage App?
*(What went well, and what could be improved?)*	(Open text field)

*Usability and Satisfaction*

In this section, we focus on the usability of the Stroke Triage App and your satisfaction with its features. Below are several statements. We ask you to indicate the extent to which you agree with each statement.

| Statement | Strongly agree (1) | Agree (2) | Neutral (3) | Disagree (4) | Strongly disagree (5) | I don’t know (6) |
| --- | --- | --- | --- | --- | --- | --- |
| Q15 I think I would like to use the Stroke Triage App frequently | ○ | ○ | ○ | ○ | ○ | ○ |
| Q16 I found the Stroke Triage App unnecessarily complex | ○ | ○ | ○ | ○ | ○ | ○ |
| Q17 I found the Stroke Triage App easy to use | ○ | ○ | ○ | ○ | ○ | ○ |
| Q18 I think I would need the help of an expert to use this system | ○ | ○ | ○ | ○ | ○ | ○ |
| Q19 I found the different functions of this system well integrated | ○ | ○ | ○ | ○ | ○ | ○ |
| Q20 I think there were too many inconsistencies in this system | ○ | ○ | ○ | ○ | ○ | ○ |
| Q21 I can imagine that most people would learn to use this system very quickly | ○ | ○ | ○ | ○ | ○ | ○ |
| Q22 I found the Stroke Triage App very difficult to use | ○ | ○ | ○ | ○ | ○ | ○ |
| Q23 I felt confident while using the Stroke Triage App | ○ | ○ | ○ | ○ | ○ | ○ |
| Q24 I had to learn a lot of things before I could get started with the Stroke Triage App | ○ | ○ | ○ | ○ | ○ | ○ |

Q25 Do you have any suggestions or areas for improvement for the Stroke Triage App?
*(For example, regarding the interface, practical usability, or additional features)*

(Open text field)

*Perceived Usefulness and Preferences*

In this section, we examine how clear, accurate, and useful you find the recommendations provided by the Stroke Triage App.

Q26 How understandable do you find the recommendations provided by the Stroke Triage App?

○ Very easy to understand
○ Easy to understand
○ Neutra
○ Difficult to understand
○ Very difficult to understand

Q27 How accurate do you find the recommendations provided by the Stroke Triage App in performing your tasks?

○ Very accurate
○ Accurate
○ Neutral
○ Inaccurate
○ Very inaccurate

Q28 Thinking about your workflow before using the Stroke Triage App, do you feel that the app has improved, worsened, or not changed your workflow?

○ Much better
○ Slightly better
○ About the same
○ Slightly worse
○ Much worse

Q29 To what extent do you experience anxiety when using the Stroke Triage App?

○ Very high: I am hesitant to use the Stroke Triage App because I am afraid of making mistakes that could have serious consequences for the patient
○ High: I am concerned that I might make an incorrect assessment when using the Stroke Triage App, which could affect patient care
○ Neutral: I do not have strong feelings of anxiety about using the Stroke Triage App
○ Low: I have few concerns about using the Stroke Triage App but remain alert and careful when filling it in
○ None: I do not feel anxious when using the Stroke Triage App and use it without hesitation

Q30 How do you experience using the Stroke Triage App within your daily work routine?

(Open text field)

Q31 How likely are you to continue using the Stroke Triage App in your daily practice in the future?

○ Very likely
○ Likely
○ Neutral
○ Unlikely
○ Very unlikely
○ I don’t know

*Sociodemographic Information*

In this section of the questionnaire, we ask a few questions about your background. This information helps us gain better insight into the diversity of our participants and allows us to interpret the study results accurately.

Q32 What is your age in years?
*(Please provide your answer in full years)*	(Open text field)

Q33 What is your gender?

○ Male
○ Female
○ Non-binary / other
○ Prefer not to say

Q34 What is your highest completed education?

○ MBO
○ HBO Bachelor
○ HBO Master
○ WO Bachelor
○ WO Master
○ Doctorate (PhD, postdoctoral, etc.)
○ Other, namely: (open text field)

Q35 How many hours per week do you work on average as a paramedic?
*(Please provide your answer in full hours)*

(Open text field)

Q36 How many years of experience do you have as a paramedic?
*(Please provide your answer in full years)*

(Open text field)

Q37 How would you rate your overall skill with medical apps and digital tools that you use in your work?

○ Expert: I can solve complex technical problems, develop or customize advanced software, and support colleagues in using technology in their work
○ Advanced: I can use advanced medical software and apps, and I am able to troubleshoot technical problems with equipment
○ Intermediate: I can use medical software and apps independently and solve simple technical problems, such as setting up notifications
○ Beginner: I only use technology for basic tasks, such as emailing and browsing the internet

Q38 How often do you use technology (e.g., smartphones, computers, tablets) in your daily life?

○ Daily
○ Regularly (3–5 times per week)
○ Occasionally (1–2 times per week)
○ Rarely (less than once per week)

Q39 How would you describe your attitude towards medical apps and digital tools?

○ Very positive: I am always looking for new technology and enjoy using it
○ Positive: I use technology often and find it useful
○ Neutral: I do not have a strong opinion about technology
○ Negative: I use technology but often encounter problems with it
○ Very negative: I avoid technology whenever possible

*Follow-up*

Would you be willing to be contacted for an interview to discuss your experiences with the Stroke Triage App in more detail?

In the interview, we will explore your experiences with the Stroke Triage App in greater depth, in order to gain better insight into your perspectives. Your personal data and responses will not be traceable back to you. The interview will be recorded and is expected to last approximately 30–45 minutes, and can be conducted either in person or online, at a time that is most convenient for you. As a token of appreciation for your participation, we will offer you a small gift or refreshment.

○ Yes, I consent to being contacted after this study to be asked whether I would like to participate in a follow-up study. I also consent to the collection and use of audio recordings. These recordings will be destroyed after the completion of the study. (-> the respondent will be redirected to a separate survey where they will be asked to provide their email address for follow-up purposes.
○ No (-> end of survey)
