# Supplementary material for: Implementation and Evaluation of the Stroke Triage App Among Paramedics in Prehospital Emergency Care: Sequential Mixed Methods Study
Source: J Med Internet Res. 2026 Nov 3;28:e105987. doi: 10.2196/105987 (PMC13735210; doi:10.2196/105987)
Supplement: Supplementary file 3 [file jmir-v28-e105987-s003.docx]

Multimedia Appendix 4. Overview of survey constructs, item characteristics, scoring, and psychometric properties.

| Variables | Source | Scoring | Example item |
| --- | --- | --- | --- |
| Demographics | By authors | 5 items, multiple choice | *How many year have you worked as a paramedic?* |
| Introduction | By authors | 2 items, multiple choice | *How did you first hear about the STA?* |
| Training | By authors | 5 items, Likert scales and open-ended question | *How relevant did you find the training for the STA?* |
| Impact on workflow | UTAUT CFIR, NASSS | 5 items, Likert scale and open-ended questions | *Has your STA use increased or decreased since the beginning? And why?* |
| Actual use | UTAUT, CFIR | 6 items, Likert scale and open-ended question | *How often do you complete all steps in the STA?* |
| Usability | D-SUS*, UTAUT | 11 items, Likert scales 0-5 | *I found the STA easy to use* |
| App accuracy and understandability | UTAUT, CFIR | 3 items, Likert scales | *Do you find the STA triage advice easy to understand?* |
| Technology effort expectancy and self-efficacy | UTAUT, NASSS | 3 items, Likert scale 0-5 | *How often do you use technology in daily life?* |
| Technology attitude and acceptance | UTAUT | 2 items, expert –to beginner, very positive to very negative ( 0 – 5) | *How would you describe your attitude towards digital health technologies compared to other digital apps?* |
| Fear | UTAUT | 1 item, no fear at all to very fearful (0 – 5) | *Do you experience any fears when using the STA?* |
| Future intention to use | UTAUT, NASSS | 1 item, very unlikely to very likely (0 - 5) | *How probable is it that you would use the STA in future clinical practice?* |

Overview of variables and measures used in the online Qualtrics survey.^1^ In total, the survey consists of 39 questions, varying from Likert scales (0-5 strongly agree- strongly disagree), multiple choice, and open-ended questions. Since some questions were subdivided into multiple questions depending on the answer given in the question prior, 42 items were eventually described in the online survey.
**Cronbach’s alpha for D-SUS: 0.74.*

References

1.	Qualtrics. The Leading Experience Management Software, version XM. 2026.
