# Supplementary material for: Implementation and Evaluation of the Stroke Triage App Among Paramedics in Prehospital Emergency Care: Sequential Mixed Methods Study
Source: J Med Internet Res. 2026 Nov 3;28:e105987. doi: 10.2196/105987 (PMC13735210; doi:10.2196/105987)
Supplement: Supplementary file 5 [file jmir-v28-e105987-s005.docx]

Multimedia Appendix 2. Good Reporting of A Mixed Methods Study (GRAMMS) **^1^** checklist

| Item |  | Elaboration | Section/ subheading |
| --- | --- | --- | --- |
| 1 | Describe the justification for using a mixed methods approach to the research question | A mixed-methods approach was used to gain a comprehensive understanding of the implementation process of the STA. Quantitative data (dashboard analytics and an online survey) provided a broad overview of usage and usability, while qualitative interviews allowed for an in-depth exploration of the paramedics' experiences and the context behind the numbers. | Study design |
| 2 | Describe the design in terms of the purpose, priority and sequence of methods | The study employed a sequential explanatory mixed-methods design. Quantitative data collection and analysis (Phase 1) were conducted first, followed by a qualitative phase (Phase 2) to further explain and contextualize the quantitative results. | Study design |
| 3 | Describe each method in terms of sampling, data collection and analysis | Sampling: Purposive and snowball sampling of paramedics in two EMS regions. Data collection: Quantitative data via app dashboard analytics and a 39-item online survey; qualitative data via semi-structured interviews. Analysis: Descriptive statistics (R Studio) for quantitative data and thematic analysis (ATLAS.ti) for qualitative data. | Quantitative phase, Qualitative phase |
| 4 | Describe where integration has occurred, how it has occurred and who has participated in it | Integration occurred during the interview guide design phase (selection of interview topics based on survey results) and at the final interpretation level. A joint display was used to merge findings from both phases to identify areas of convergence, complementarity, or dissonance. | Data analysis and integration |
| 5 | Describe any limitation of one method associated with the present of the other method | The quantitative phase was limited in explaining the "why" behind low usage rates despite high usability scores. While the qualitative phase addressed this, the sequential design may have introduced participation bias, as paramedics who were more engaged or held stronger opinions in the first phase were more likely to participate in the interviews. | Study design, Discussion |
| 6 | Describe any insights gained from mixing or integrating methods | Mixing methods revealed that while the STA was rated as highly usable quantitatively, the qualitative data highlighted critical context-specific barriers, such as perceived time pressure and the need for better integration with existing EMS workflows, which the survey alone could not fully capture. | Main results, Discussion |

References

1.	O'Cathain A, Murphy E and Nicholl J. The quality of mixed methods studies in health services research. *J Health Serv Res Policy* 2008; 13: 92-98. 2008/04/18.
